# Gallbladder agenesis leading to an unnecessary surgery: a case report

**DOI:** 10.3389/fmed.2025.1714980

**Published:** 2025-12-11

**Authors:** Zizhen Yin, Yu Liu

**Affiliations:** 1 Department of Hepatobiliary Surgery, Heze Medical College, Heze, Shandong, China; 2 Department of Internal Medicine, Central Hospital in Mudan District of Heze, Heze, Shandong, China

**Keywords:** gallbladder agenesis, cholecystectomy, laparoscopy, abdominal pain, magnetic resonance cholangiopancreatography

## Abstract

**Background:**

Gallbladder agenesis is a rare congenital anomaly of the biliary system. Preoperative diagnosis of gallbladder agenesis is challenging and such conditions are often identified intraoperatively. In this report, we present a case of gallbladder agenesis encountered during laparoscopic cholecystectomy.

**Case presentation:**

A 47-year-old female patient attended our hospital in August 2023 due to distension and discomfort in the right upper quadrant. Ultrasound images revealed that the patient’s gallbladder was filled with gallstones. The patient was admitted to our hospital for laparoscopic cholecystectomy; however, no gallbladder was visualized during the laparoscopy, and the final diagnosis was congenital absence of the gallbladder.

**Conclusion:**

The diagnosis of Gallbladder agenesis remains a challenge, It may lead to an unnecessary surgery if physicians do not pay attention to careful differential diagnosis.

## 1 Introduction

Gallbladder agenesis is a rare congenital anomaly characterized by the absence of gallbladder development, which is seldom encountered in surgery, particularly during laparoscopic cholecystectomy. This condition is frequently overlooked by physicians, posing significant diagnostic challenges. Despite significant advances in imaging technology, the preoperative diagnosis of gallbladder agenesis remains difficult. In August 2023, a case of congenital absence of the gallbladder was encountered during laparoscopic cholecystectomy in our hospital. The patient was misdiagnosed and lead to an unnecessary surgery, we present this case for the benefit of colleagues.

## 2 Case presentation

**Figure 1:**
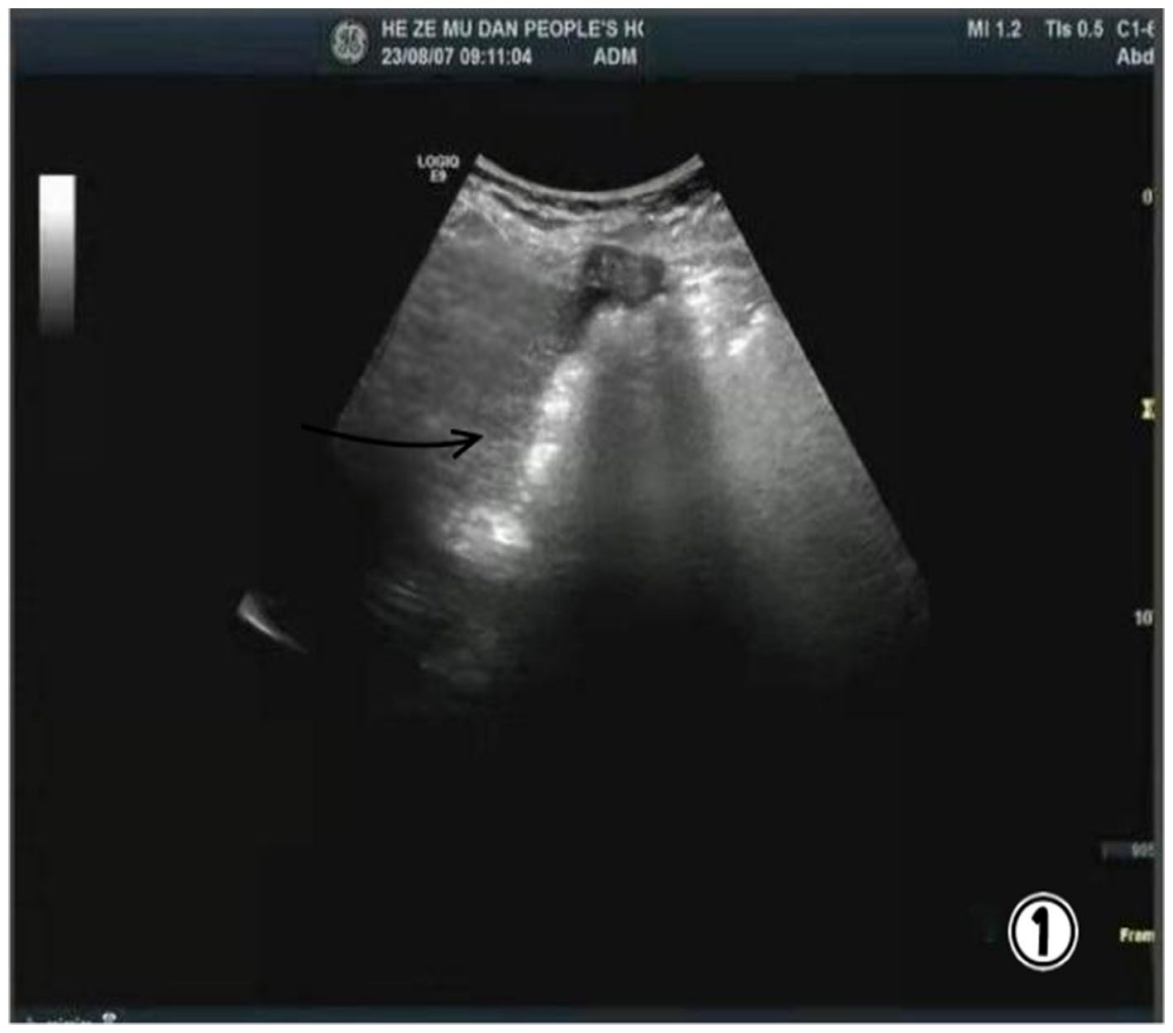
Ultrasound image showing gallstones.

A 47-year-old woman presented with a 9-month history of discomfort in the right upper abdomen, which was exacerbated by the consumption of fatty foods. She had previously undergone an ultrasound examination at another hospital, where she was diagnosed with gallstones and chronic cholecystitis 9 months ago. Subsequent ultrasound evaluation at our facility confirmed that her gallbladder remained replete with gallstones (Figure 1). Due to the patient’s abdominal discomfort and ultrasound evidence, she was admitted to hospital for laparoscopic cholecystectomy.

**Figure 2:**
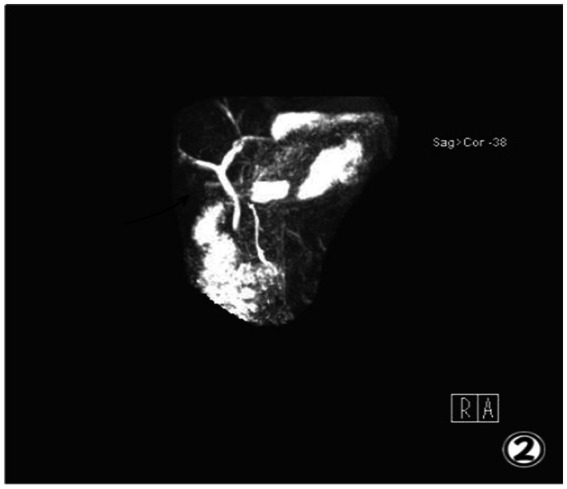
MRCP image showed no gallbladder.

The patient had no history of heart disease, hypertension, diabetes, jaundice, fever or other diseases. The patient did not have any relevant family medical history. The right upper abdomen exhibited slight tenderness with no other positive signs. The patient underwent blood tests, including a complete blood count, C-reactive protein, electrolytes, and tests to evaluate liver, kidney, and pancreas function. All liver function tests including aspartate aminotransferase (19.9 U/L), alkaline phosphatase (76.2 U/L), alanine aminotransferase (15.4 U/L), *γ*-glutamy transferase (8.1 U/L), and total bilirubin (5.3 umol/L) were normal. The white blood cell count (8.33 × 10^9^/L) and C-reactive protein level (5 mg/L) were within normal limits. Additionally, renal and pancreatic function tests, as well as electrolyte levels, were all within normal ranges. Magnetic resonance cholangiopancreatography (MRCP) was conducted; however, no images of the gallbladder were obtained, It was determined that the gallbladder was full of gallstones which prevented it from being imaged (Figure 2).

**Figure 3:**
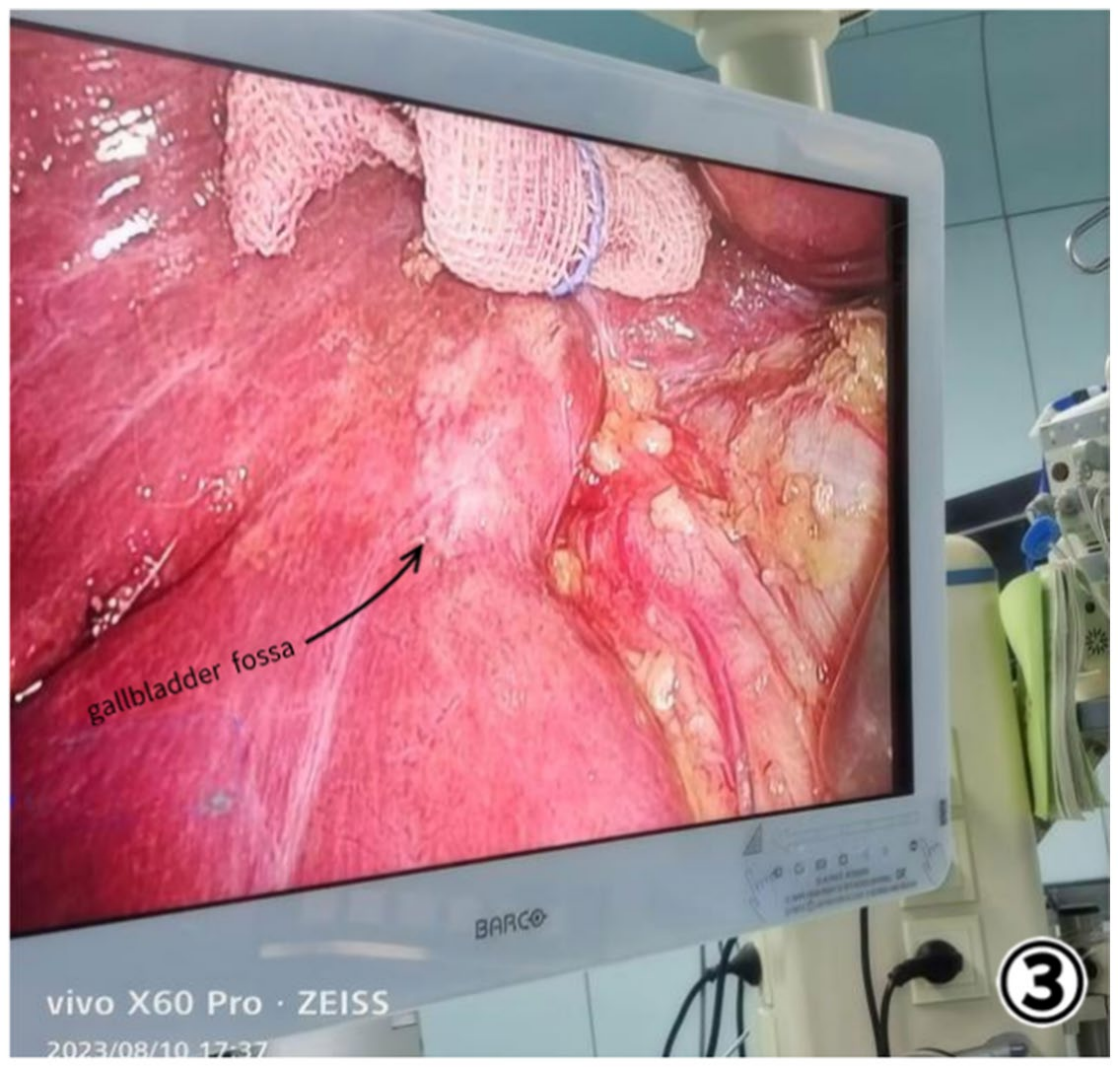
No gallbladder was visualized.

**Figure 4:**
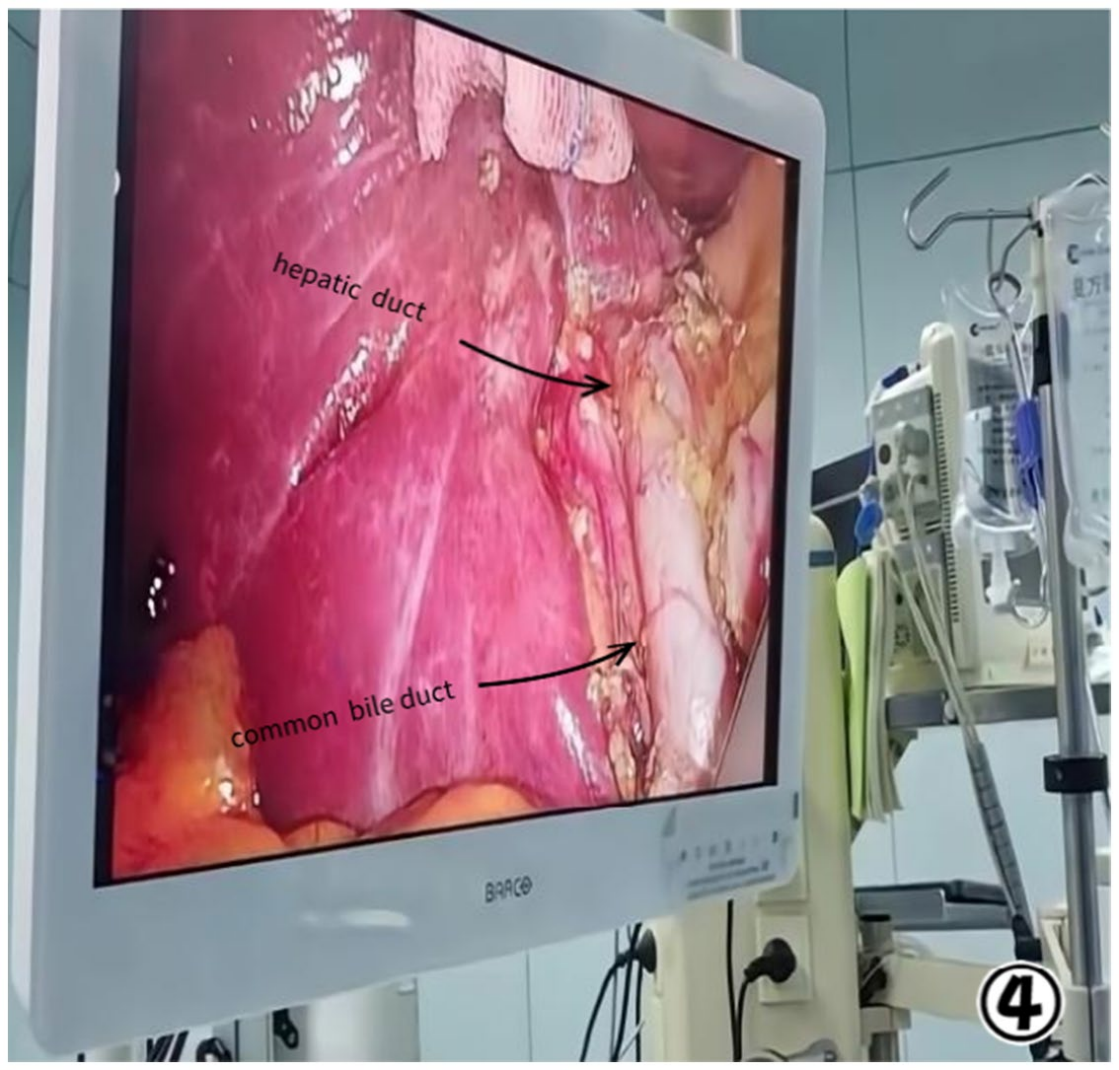
No cystic duct was visualized.

The patient was diagnosed with gallstones and chronic cholecystitis, contingent on the ultrasound diagnosis and MRCP analysis, the patient underwent surgery 3 days after admission. We were surprised to find the gallbladder fossa completely empty during laparoscopic examination. To ascertain the location of the gallbladder and rule out the possibility of an ectopic gallbladder, intraoperative ultrasonography was conducted; however, no gallbladder could be detected either outside or within the liver parenchyma. Subsequently, we performed a meticulous dissection and exposure of both the common bile duct and hepatic duct, yet neither the gallbladder nor cystic duct was visualized (Figures 3, 4). Congenital agenesis of the gallbladder was confirmed intraoperatively, leading us to terminate the operation.

We adopted a conservative management approach utilizing smooth muscle relaxants. The patient responded well to pain control and antispasmodics. There was a notable alleviation of distension and discomfort in the right upper quadrant. The patient ultimately achieved a satisfactory recovery after laparoscopy and was subsequently discharged from the hospital. Postoperative computed tomography scans consistently showed no evidence of a gallbladder.

We conducted follow-up assessments at 3 months, 6 months, and 1 year postoperatively. Throughout this period, her liver function remained stable without any significant abnormalities. Postoperative follow-up indicated that the patient had successfully returned to work, experienced no further right upper abdominal pain, and exhibited no notable changes in her diet.

## 3 Discussion

We encountered a case of gallbladder agenesis during laparoscopic cholecystectomy, although preoperative ultrasound images indicated that the gallbladder was filled with gallstones, neither the gallbladder nor the cystic duct could be visualized during laparoscopic cholecystectomy. Our patient had been misdiagnosed and lead to an unnecessary surgical intervention. This situation highlighted our erroneous preoperative diagnosis. Herein, we present this case and conduct a comprehensive review of the literature on gallbladder agenesis, along with an analysis of the underlying factors that contributed to our diagnostic errors.

Congenital absence of the gallbladder is a rare congenital anomaly of the biliary system, with an estimated incidence of 10–65 per 100,000 (1). The pathogenesis of gallbladder agenesis is known to be related to embryonic development (2). The gallbladder is currently recognized to develop from the hepatic diverticulum and the liver primordium. Initially, it forms as a hollow bud that arises from the duodenum (3). This bud subsequently divides, with one portion differentiating into the gallbladder and cystic duct, while the other portion gives rise to the glandular tissue of the liver (3). A failure in this division or an inability of the solid bud to become hollow at the 5th week of gestation can result in agenesis of the gallbladder (4, 5). This condition was first reported by Lemery in 1701 (6), and to date, approximately 500 cases have been documented worldwide (7, 8), Gallbladder agenesis is typically discovered during surgical procedures or autopsies (9). Some patients may be asymptomatic (10–12), whereas others exhibit symptoms. The most common symptom reported is pain in the right upper quadrant (13, 14), with additional symptoms including intolerance to fatty foods, flatulence and indigestion (15).

It is uncertain why patients experience upper abdominal pain. Some researchers believe that the right upper abdominal pain experienced by patients may be caused by spasms of the Oddi sphincter (16, 17). Some researchers have also suggested that the cause of pain in patients with gallbladder agenesis is similar to the post-cholecystectomy syndrome pain which arises from the dilation of the common bile duct as it attempts to store bile (18). This condition leads to increased pressure within the sphincter of Oddi (19). Conservative management can effectively alleviate the associated symptoms, and surgical exploration can be avoided if a precise diagnosis is established prior to surgery (20).

Many physicians remain unfamiliar with gallbladder agenesis. Most cases are typically identified during surgical procedures (21, 22), Some cases have been recorded in conjunction with cholecystectomy (23, 24), in those cases, surgical intervention were performed based solely on the results of ultrasound examination, leading to unnecessary surgery and a high intraoperative incidence of gallbladder agenesis (25–27). If the gallbladder is not found during surgery, physicians must rule out an ectopic gallbladder by exploration although this meticulous surgical exploration may result in bile duct injury and increase the need to convert to open access (28–30).

Abdominal ultrasound serves as the primary diagnostic tool for evaluating gallbladder diseases (31), with an accuracy rate of 95% (32), but in cases of congenital malformations, the sensitivity of abdominal ultrasound decreases to 61% (33). MRCP was regarded as the gold standard for evaluating this condition (17, 18), it serves as an excellent complement in some cases with indeterminate findings. Cholangiography may be a good method for definitive diagnosis in such situations to prevent unnecessary bile duct injury caused by excessive operative exploration (19, 34). The result of a hepatobiliary iminodiacetic acid scan and endoscopic retrograde cholangiopancreatography may be misleading in cases of gallbladder agenesis (35), non-visualization of the gallbladder is more likely to be misinterpreted as obstruction of the cystic duct, which aligns with a diagnosis of cholecystitis, rather than being recognized as absence of the gallbladder (35).

In this instance, ultrasound examinations indicated the presence of gallstones, but no MRCP images of gallbladder were visualized, at that time, we mistakenly assumed that it was filled with gallstones, which prevented it from being imaged. When the imaging findings appear contradictory, we had not employed a complementary method to clarify the diagnosis, but proceeded with laparoscopy blindly, the possibility of gallbladder agenesis was overlooked at that time. Subsequently, the gallbladder fossa was found to be completely empty during surgery, although intraoperative ultrasonography and a meticulous dissection was conducted, neither the gallbladder nor the cystic duct could be identified. Gallbladder agenesis was confirmed intraoperatively, postoperative computed tomography scans consistently confirmed the absence of the gallbladder.

The patient was misdiagnosed, ultimately resulting in an unnecessary surgical intervention. The misdiagnosis resulted from low clinical suspicion and the high false-positive rate associated with ultrasound imaging. Ultrasonography is highly susceptible to interference from intestinal gas, potentially leading to misdiagnosis due to artifacts caused by the intestinal gas, this interference may have resulted in the erroneous detection of gallstones (36). Specifically, a gas-filled duodenum that contains reflective food particles can mimic the appearance of a stone-filled gallbladder (21). The consecutive misdiagnoses across two institutions highlight systemic challenges in recognizing this rare condition.

This case was misdiagnosed primarily due to over-reliance on ultrasound findings and misjudgment of MRCP without confirmatory imaging or comprehensive evaluation, and cognitive biases toward more common pathologies, and insufficient awareness of gallbladder agenesis as a diagnostic entity. Physicians are prone to cognitive biases when evaluating patients with right upper abdominal pain, often prioritizing common conditions such as cholecystitis and cholelithiasis, thereby neglecting rarer etiologies.

In this instance, the ultrasound report was accepted without critical evaluation, MRCP findings were misinterpreted, and appropriate supplementary investigations were not pursued, it is a valuable lesson to be learned. To mitigate these cognitive biases, physicians should expand their diagnostic considerations when imaging findings appear contradictory. For patients exhibiting atypical symptoms without abnormal findings on routine examinations, rare conditions such as congenital absence of the gallbladder should be taken into account.

This case serves as an important reminder for surgeons and radiologists to be vigilant and consider differential diagnoses related to gallbladder absence when imaging discrepancies arise, particularly when MRCP fails to visualize the gallbladder alongside ultrasound findings suggesting cholelithiasis. In such scenarios, differential diagnosis must systematically consider the following conditions: severe gallbladder atrophy, complete stone filling causing non-visualization, heterotopic gallbladder, hypoplastic gallbladder, and congenital gallbladder absence.

Artificial intelligence has recently been introduced in laparoscopic cholecystectomy surgeries (37). It appears as a promising tool, enabling physicians to safely dissect during laparoscopic cholecystectomy. it can provide critical view of safety to distinguish gallbladder agenesis and avoid bile duct injuries (38). Nonetheless, conservative management can effectively alleviate the associated symptoms of gallbladder agenesis and surgical exploration can be avoided. When the diagnosis remains uncertain, it is not advisable to proceed with blind exploratory surgery.

## 4 Conclusion

Despite significant advancements in imaging technology, gallbladder agenesis remains frequently misdiagnosed prior to surgery. Conservative treatment strategies are effective, when the imaging findings appear contradictory, over-reliance on any single examination without appropriate supplementary investigations and insufficient awareness of gallbladder agenesis may lead to misdiagnosis and an unnecessary surgery.

## Data Availability

The raw data supporting the conclusions of this article will be made available by the authors, without undue reservation.
